# A Machine Learning Approach in Autism Spectrum Disorders: From Sensory Processing to Behavior Problems

**DOI:** 10.3389/fnmol.2022.889641

**Published:** 2022-05-09

**Authors:** Heba Alateyat, Sara Cruz, Eva Cernadas, María Tubío-Fungueiriño, Adriana Sampaio, Alberto González-Villar, Angel Carracedo, Manuel Fernández-Delgado, Montse Fernández-Prieto

**Affiliations:** ^1^Centro Singular de Investigación en Tecnoloxías Intelixentes da USC (CiTIUS), Universidade de Santiago de Compostela (USC), Santiago de Compostela, Spain; ^2^The Psychology for Positive Development Research Center, Lusíada University—North, Porto, Portugal; ^3^Genomics and Bioinformatics Group, Centre for Research in Molecular Medicine and Chronic Diseases (CiMUS), Universidade de Santiago de Compostela (USC), Santiago de Compostela, Spain; ^4^Grupo de Medicina Xenómica, Universidade de Santiago de Compostela (USC), Santiago de Compostela, Spain; ^5^Fundación Instituto de Investigación Sanitaria de Santiago de Compostela (FIDIS), Santiago de Compostela, Spain; ^6^Psychological Neuroscience Lab, Centro de Investigação em Psicologia, School of Psychology, University of Minho, Campus de Gualtar, Braga, Portugal; ^7^Fundación Pública Galega de Medicina Xenómica, Servicio Galego de Saúde (SERGAS), Santiago de Compostela, Spain; ^8^Grupo de Medicina Xenómica, U-711, Centro de Investigación en Red de Enfermedades Raras (CIBERER), Universidade de Santiago de Compostela (USC), Santiago de Compostela, Spain; ^9^Grupo de Genética, Instituto de Investigación Sanitaria de Santiago (IDIS), Santiago de Compostela, Spain

**Keywords:** machine learning, autism spectrum disorders, sensory processing, behavior problems, regression

## Abstract

Atypical sensory processing described in autism spectrum disorders (ASDs) frequently cascade into behavioral alterations: isolation, aggression, indifference, anxious/depressed states, or attention problems. Predictive machine learning models might refine the statistical explorations of the associations between them by finding out how these dimensions are related. This study investigates whether behavior problems can be predicted using sensory processing abilities. Participants were 72 children and adolescents (21 females) diagnosed with ASD, aged between 6 and 14 years (M = 7.83 years; SD = 2.80 years). Parents of the participants were invited to answer the Sensory Profile 2 (SP2) and the Child Behavior Checklist (CBCL) questionnaires. A collection of 26 supervised machine learning regression models of different families was developed to predict the CBCL outcomes using the SP2 scores. The most reliable predictions were for the following outcomes: total problems (using the items in the SP2 touch scale as inputs), anxiety/depression (using avoiding quadrant), social problems (registration), and externalizing scales, revealing interesting relations between CBCL outcomes and SP2 scales. The prediction reliability on the remaining outcomes was “moderate to good” except somatic complaints and rule-breaking, where it was “bad to moderate.” Linear and ridge regression achieved the best prediction for a single outcome and globally, respectively, and gradient boosting machine achieved the best prediction in three outcomes. Results highlight the utility of several machine learning models in studying the predictive value of sensory processing impairments (with an early onset) on specific behavior alterations, providing evidences of relationship between sensory processing impairments and behavior problems in ASD.

## Introduction

Autism spectrum disorder (ASD) is a neurodevelopmental condition with a consistently high prevalence worldwide (Chiarotti and Venerosi, 2020) that poses a serious burden to the society and affected families. An early diagnosis of ASD is crucial for implementing interventional approaches on individuals with this disorder (Cidav et al., 2017). Multiple risk factors contribute to the ASD phenotype, including genetic, biological, psychosocial, and environmental contributors (Parenti et al., 2021; Deb and Bateup, 2022). This disorder is characterized by impairments in different areas of development, including deficits in social interactions and communication, by restricted/repetitive behaviors, and by sensory-perceptual alterations, which were added as an ASD diagnostic criterion (American Psychiatric Association, 2013). Impairments in sensory processing are described as unusual interests in sensory aspects of the environment (e.g., visual, auditory, or tactile stimuli) to which individuals with ASD frequently display atypical responses (Tseng et al., 2011; American Psychiatric Association, 2013). These responses can be organized and classified as hyper-reactivity (tendency to respond at lower intensity thresholds quicker and more intensely or longer) or hypo-reactivity (tendency to respond with “indifference,” unawareness, or slowly) to sensory input (Tavassoli et al., 2016) that seems to be underpinned by specific neurophysiological markers (see Marco et al., 2011 for a detailed review). Atypical processing patterns have been observed in ASD across all sensory modalities, including visual, auditory, olfactory, proprioceptive, somatosensory, or interoceptive stimulation, and multisensory integration, regardless of age and symptoms severity in children and adolescent (Ben-Sasson et al., 2009). Sensory alterations have an early onset and are one of the first signs of ASD, as early as observed in the first months of life (Iarocci and McDonald, 2006). In consequence, these alterations can impact behavior and social functioning of children and adolescents and may be at the root of social deficits during development (Thye et al., 2018).

Indeed, altered sensory responsivity in ASD cascades into social and behavioral impairments (Thye et al., 2018). The relationship between hypo-responsiveness and hyper-responsiveness to sensory stimuli and maladaptive behavior has been documented, with atypical processing of stimuli being correlated with social, cognitive, and communicative impairments (Kojovic et al., 2019), and the presence of repetitive and restricted interests and behaviors (Foss-Feig et al., 2012). Sensory abnormalities were also linked to isolation, reactivity to change, disinterest and indifference, self-aggression, irritability, or emotional lability (Gonthier et al., 2016). Additionally, altered sensory processing has been related to anxiety (Uljarević et al., 2016) and depressive (Bitsika et al., 2016) states in children and adolescents with ASD, with a great impact on their adaptive behaviors (Tomcheck and Dunn, 2007; Lane et al., 2010; Zachor and Curatolo, 2014). One of the earliest and most common sensory alterations described in ASD is related to abnormalities in tactile processing, for example, food texture (Mikkelsen et al., 2018).

Tactile contact is considered a precursor for the development of social and communication abilities, and impaired touch processing has been linked to emotional and social distress early in life, as it imposes limits on environmental learning opportunities (Mikkelsen et al., 2018). Moreover, evidence suggests that increased difficulties in touch processing are associated with behavioral impairments (e.g., difficulties in inhibitory control) in children and adolescents with ASD (Puts et al., 2014; Piccardi et al., 2021). In addition, altered touch processing was related to increased social and communication deficits (Foss-Feig et al., 2012; Miguel et al., 2017), to non-verbal communication impairments and repetitive behaviors (Foss-Feig et al., 2012), as well as anxious/depressed states and repetitive/obsessive behavior (Fernández-Prieto et al., 2021). Therefore, it is important to further clarify the relationship between sensory processing and behavior in children and adolescents with ASD, by exploring to what extent sensory processing is predictive of behavioral outcomes in this population. The Sensory Profile 2—SP2 (Dunn, 2014)—and the Child Behavior Checklist—CBCL (Achenbach and Rescorla, 2001)—are two widely used tools to measure sensory and behavioral competencies, respectively. These questionnaires provide standardized measures of child's development and offer guidance for future clinical interventions. In addition, the scores of SP2 and CBCL subscales are correlated (Miguel et al., 2017). New tools—like machine learning (ML) techniques—can offer novel insights into how sensory processing and behavior—measured by these questionnaires—are associated. Some works in the literature showed the interest of using ML in the study of ASD to analyze continuous/dimensional or categorical/qualitative variation between and within individuals (Lombardo et al., 2019). In addition, ML can offer improved diagnostic timing, precision, and quality, allowing clinicians to provide more robust diagnosis and intervention programs (Thabtah, 2019), as well as providing evidence on possible altered processes (e.g., reactivity to sensorial stimuli) for implementing early personalized care therapies and/or strategies. The ML models provide satisfactory solutions to medical and non-medical applications due to its ability to extract information and make predictions (Briscoe and Marín, 2020).

This study aims to use a wide collection of supervised ML algorithms (multiple linear regression, support vector machines, gradient boosting machine, and regression tree-based ensembles such as cubist and random forest, among others) to investigate how sensory processing predicts behavioral problems in ASD. With these algorithms, we sought to examine how SP2 quadrant scores (seeking, avoiding, sensitivity, and registration) and touch processing (given the evidence suggesting that tactile impairments are related to the onset of ASD, and being the most common sensory alteration in this population) are predictive of CBCL subscales: anxious/depressed, withdrawn/depressed, somatic complaints, social problems, thought problems, attention problems, rule-breaking behavior, aggressive behavior, and the two empirically derived internalizing and externalizing broadband scales. It is expected that hyper-responsive and hypo-responsive sensory experiences assessed using the SP2, i.e., items corresponding to seeking and avoiding quadrants, as well as touch processing, can be used to predict ASD behavioral problems, namely, anxious/depressed, withdrawn/depressed, rule-breaking behavior and aggressive behavior, as well as in internalizing and externalizing domains.

## Materials and Methods

### Participants

Participants were 72 children and adolescents (21 females), aged between 6 and 14 years (mean = 7.83 years; SD = 2.80 years), diagnosed with ASD following the criteria established by the Diagnostic and Statistical Manual of Mental Disorders in its revised fourth version (DSM-IV-TR) or fifth version (DSM-5). Participants were part of a larger research project studying the association between phenotype and genotype characteristics in ASD. Qualified clinicians who were part of the research team confirmed the ASD diagnosis for research purposes, using the Autism Diagnostic Interview Revised, ADI-R (Rutter et al., 2006) and the Autism Diagnostic Observation Schedule, ADOS (Lord et al., 2008), both described in Sections Autism Diagnostic Interview Revised and Autism Diagnostic Observation Schedule−2, respectively, of the Supplementary Material. All parents who agreed to voluntarily participate gave the written informed consent, obtained in accordance with the Declaration of Helsinki.

### Dataset Description

The Sections Sensory Profile−2 and Child Behavior Checklist of the Supplementary Material describe the SP2 (Dunn, 2014) and CBCL (Achenbach and Rescorla, 2001) questionnaires in detail, respectively. The six sets of items from SP2—touch processing, the four quadrants (seeking, avoiding, sensitivity, and registration) and the total SP2 score (all the items)—were used as inputs of the ML models for predicting the following 11 outcomes of the CBCL questionnaire (scale scores): withdrawn/depressed (S1), somatic complaints (S2), anxious/depressed (S3), social problems (S4), thought problems (S5), attention problems (S6), rule-breaking behavior (S7), aggressive behavior (S8), internalizing problems (internal), externalizing problems (external), and total problems (total). Raw scores for each scale were converted to standardized T-scores based on Spanish standards (Unitat d'Epidemiologia i de Diagnòstic en Psicopatologia del Desenvolupament, 2016), bounded between 0 and 100, considering age and sex of participants (6–11 and 12–18 years, separately). Table 1 depicts the list of items included in each set. Each ML model predicts one of the 11 outcomes in CBCL using one of the six input sets in SP2, giving a total of 6·11 = 66 datasets. The influence of gender (denoted as G) was also investigated, as recent evidence (Osório et al., 2021) revealed that ASD females are more likely to be distressed by environmental stimuli, such as noise, and have more difficulties with movement coordination and postural control. Therefore, each set was analyzed with and without gender, thus obtaining 66·2 = 132 datasets. Supplementary Figure 1 draws the boxplots that represent the distributions of values for the 11 outcomes. Although each outcome is a score on a scale from 1 to 100, the values are above 30–40 for all the outcomes and, in several cases, they do not reach 100. For each CBCL outcome, a value below 60 corresponds to a normative behavior, values between 60 and 70 correspond to preclinical behavior (i.e., near to require clinical treatment), and values above 70 correspond to a clinical diagnosis.

**Table 1:** List of the six item sets used as inputs by the machine learning regressors (touch, total, and the four quadrants from the SP2 questionnaire), with the number of items and the items included in each set.

**Item set**	**No. items**	**Items in SP2 questionnaire**	
Touch	11	16–26	
Seeking	19	14, 21, 22, 25, 27, 28, 30, 31, 32, 41, 48, 49, 50, 51, 55, 56, 60, 82, 83	
Avoiding	20	1, 2, 5, 15, 18, 58, 59, 61, 63, 64, 65, 66, 67, 68, 70, 71, 72, 74, 75, 81	
Sensitivity	19	3, 4, 6, 7, 9, 13, 16, 19, 20, 44, 45, 46, 47, 52, 69, 73, 77, 78, 84	
Registration	22	8, 12, 23, 24, 26, 33, 34, 35, 36, 37, 38, 39, 40, 53, 54, 57, 62, 76, 79, 80, 85, 86	
Total	86	1–86	
**CBCL outcome**			
S1	Anxious/depressed	S7	Rule-Breaking behavior
S2	Withdrawn/depressed	S8	Aggressive behavior
S3	Somatic complaints	Internal	Internalizing problems
S4	Social problems	External	Externalizing problems
S5	Thought problems	Total	Total problems
S6	Attention problems		

*The lower part lists the 11 scales from the CBCL questionnaire (outcomes) to be predicted by the regressors*.

### Machine Learning

The description of the ML regression models used to predict the CBCL outcomes automatically using the SP2 scores is detailed in Section Machine Learning Techniques of the Supplementary Material, whose Supplementary Tables 1, 2 list the details of these models, grouped by families. The family of linear and regularized regressors include simple linear regression (Chambers, 1992), namely, lm, lreg, lmreg, and pylm^1^; stochastic gradient descent (sgd); least absolute shrinkage and selection operator regression (lasso) and elasticnet (enet). Kernel and support vector regressors include kernel ridge regression (krr); ε-support vector regression (Chang and Lin, 2011) with radial basis function (RBF), svr and pysvr, and linear (lsvr) kernels; and Gaussian process regression (Rasmussen and Williams, 2006), named gpr. We also used the regression tree (named tree) and the M5 model tree (Cohen, 1995), named m5, implemented by the Weka library (Frank et al., 2016). The ensemble family includes bagging (Breiman, 1996); adaboost (Drucker, 1997); gradient boosting machine (Ridgeway, 1999), named gbm and pygbm; boosting of regression trees (Wang, 2011), named bstTree; cubist (Quinlan, 1992); random forest (Breiman, 2001), named rf and pyrf; extremely randomized regression trees (extraTrees); and voting committee (vote). We also used the multilayer perceptron neural network (pymlp). The models were selected due to their high performance in our exhaustive comparison (Fernández-Delgado et al., 2019), being implemented in the Python programming language with the Scikit-learn module (Pedregosa et al., 2011), in the R statistical and computing language (The R Team, 2022), and in the Octave (Eaton et al., 2022) and MATLAB (Mathworks, 2021) scientific programming languages.

### Experimental Methodology

The classical K-fold cross-validation methodology, which splits the available dataset in training and test sets, is normally used to test the performance of ML models. The performance measurements commonly used in regression problems are the Pearson correlation coefficient (*R*), root mean squared error (RMSE), mean absolute error (MAE), and weighted absolute percentage error (WAPE), defined in Section Performance Measurements of the Supplementary Material. The reduced number (*N* = 72) of participants might lead to poorly significant training set and unreliable predictions using the K-fold cross validation with *K* = 4, 5, or 10. To avoid this drawback, we used leave-one-out cross validation, that is, a particular case of K-fold for *K* = *N*. In the *i*-th trial, with *i* = 1𠈆 *N*, the *i*-th participant is left for testing, and the remaining *N*-1 participants are used to select adequate hyper-parameter values that achieve good performance (hyper-parameter tuning). The regressor is trained on a subset of the *N*-1 participants (training set) using a given combination of hyper-parameter values. The remaining participants (validation set) evaluate the regressor performance using that combination of values. In order to avoid biasing caused by splitting, the *N*-1 participants are sorted by increasing outcome value and participants with odd (even) index are assigned to the training (validation) set. Thus, each set roughly contains 50% of the *N*-1 participants with outcome values distributed across the whole range. The training/validation cycle is repeated for the different combinations of hyper-parameter values, and the combination with the lowest RMSE on the validation set is selected. Finally, the regressor is trained with both sets (training and validation, summing up *N*-1 participants), using the selected combination of hyper-parameter values, and tested on the *i*-th participant, which was devoted to testing. The tuning/testing process is repeated for each trial *i* = 1,…, *N*, and the performance, measured by *R*, RMSE, MAE, and WAPE, is calculated over the *N* trials. The same process is executed for each model and CBCL outcome.

To measure the validity of the prediction to perform a diagnosis, each CBCL outcome is thresholded (see Section Dataset Description) and labeled as normative (preclinical or clinical) with values below (above) 60. The usual performance measurements are accuracy (ACC), sensitivity (Se), and specificity (Sp), defined in Section Performance Measurements of the Supplementary Material. This classification problem is not intended to replace the original prediction of the continuous CBCL outcome, but to estimate the impact of unreliable predictions on a final diagnostic (normative vs. preclinical and clinical).

## Results

Table 2 summarizes the best correlation *R* achieved by some regressors for each CBCL outcome (in columns) and the set of SP2 items (input, in rows) with and without gender. Each *R* value is the correlation between the true CBCL outcome values and the values predicted by the regressor for this outcome over the *N* patients, according to the abovementioned leave-one-out approach. These *R* values are not the correlations between CBCL outcomes and SP2 items. Table 3 reports the best correlation *R*, and its corresponding RMSE, MAE, and WAPE values (in %), achieved by the best regressor for each outcome with and without gender (G), alongside the set of SP2 items that provided the best *R*. The last three rows of Table 3 report the accuracy, sensitivity, and specificity classification.

**Table 2:** The best *R* for each outcome over the scales is in bold, labeled by performance levels as: BTM (bad to moderate), MTG (moderate to good), and VGE (very good to excellent).

	**CBCL outcome**										
**Item set**	**S1**	**S2**	**S3**	**S4**	**S5**	**S6**	**S7**	**S8**	**Internal**	**External**	**Total**
Touch	0.54	0.3	0.21	0.68	**0.57** **MTG**	0.5	**0.44** **BTM**	0.47	0.49	0.4	**0.67** **MTG**
Seeking	0.11	0.04	**0.44** **BTM**	0.34	0.39	**0.51** **MTG**	0.29	0.48	0.16	0.42	0.34
Avoiding	**0.72** **MTG**	0.57	0.28	0.49	0.26	0.23	0.39	**0.55** **MTG**	**0.63** **MTG**	0.54	0.56
Sensitivity	0.43	0.36	0.2	0.51	0.41	0.4	0.35	0.36	0.43	0.37	0.47
Registration	0.69	**0.58** **MTG**	0.35	**0.72** **MTG**	0.38	0.43	0.32	0.48	**0.63** **MTG**	0.42	0.58
Total	0.68	0.41	0.36	0.63	0.51	0.43	0.3	0.5	0.61	**0.98** **VGE**	0.57

*Best correlation (R) between true and predicted value for each CBCL outcome (in columns) and for each SP2 scale (touch, seeking, avoiding, sensitivity, registration, and total, in rows)*.

**Table 3:** Correlation (*R*), RMSE, MAE, WAPE (in %), and input set that achieved the best performance for each CBCL outcome.

**Outcome**	**S1**	**S2**	**S3**	**S4**	**S5**	**S6**	**S7**	**S8**	**Internal**	**External**	**Total**
*R*	0.72	0.58	0.44	0.72	0.57	0.51	0.44	0.55	0.63	0.98	0.67
RMSE	8.56	16.30	10.05	9.93	21.98	8.67	9.46	12.05	9.89	2.32	9.16
MAE	6.39	12.44	7.71	8.05	18.15	6.6	7.42	9.12	7.79	1.07	7.07
WAPE (%)	11.9	18.93	14.22	11.9	25.8	10.1	13.6	15.95	13.18	1.86	11.20
Input set	Avoid	Reg+G	Seek	Reg	Touch	Seek+G	Touch+G	Avoid+G	Avoid	Total	Touch
Regressor	adaboost	pymlp	pygbm	pygbm	lreg	pyrf	extraTrees	tree	ridge	lm	pygbm
Acc (%)	87.5	70.8	79.2	66.7	70.8	77.8	77.8	73.6	79.2	97.2	73.6
Se (%)	56.2	77.5	41.2	82.9	68	87.7	35.7	54.2	78.6	100	78.4
Sp (%)	81.8	72.1	58.3	66.7	87.2	84.7	41.7	61.9	71	92.3	72.5

*Besides, accuracy, sensitivity, and specificity (in %) and best regressor for the classification into normative range vs. pre-clinical and clinical*.

According to the Colton criteria in Section Performance Measurements of the Supplementary Material, the prediction (Table 2) for externalization domain (external outcome, *R* = 0.98) is considered “very good to excellent” (labeled VGE in the table), while the prediction of anxious/depressed (S1) and social problems (S4), with *R* = 0.72, is “moderate to good” (MTG) but close to “very good to excellent.” The prediction of withdrawn/depressed (S2), thought problems (S5), attention problems (S6), aggressive behavior (S8), internalizing and total problems is also “moderate to good” (MTG). Finally, the predictions of somatic complaints (S3) and rule-breaking (S7) behaviors are “bad to moderate” (BTM), and therefore fairly unreliable.

The avoiding quadrant is the most reliable in anxious/depressed (S1), aggressive behavior (S8), and internalizing problems as shown in Table 2. The registration quadrant best predicts withdrawn/depressed (S2), social problems (S4), and internalizing (same *R* as avoiding). Touch processing best predicts thought problems (S5), rule-breaking (S7, with low *R* = 0.44), and total problems. The seeking quadrant achieves low *R* = 0.44 in somatic complaints (S3) and *R* = 0.51 in attention problems (S6), so it is not very related to any of the problems. The sensitivity quadrant never gets the best reliability in prediction. The influence of gender on the prediction is marginal, with very small differences (about 0.02) in the best *R* for each outcome with and without gender, although this influence might be implicit on the remaining items of the questionnaire.

Table 3 lists the best *R* results, extracted from Table 2, alongside their corresponding RMSE, MAE, and WAPE values, best SP2 quadrant and regressor, and accuracy, sensitivity, and specificity of normative vs. preclinical and clinical classification. The MAE is below 10 for all the CBCL outcomes, except withdrawn/depressed (S2) and thought problems (S5), so the uncertainty prediction value for most outcomes is below 20. The outcome values range approximately between 35 and 100 (see Supplementary Figure 1), so these MAE values are comparatively small. The WAPE is very low in externalizing problems (1.86%) and below 14% for most scales except withdrawn/depressed (S2), somatic complains (S3), thought problems (S5), and aggressive behavior (S8).

The pygbm (gradient boosting machine) is the regressor that achieves the best *R* in more (three) CBCL outcomes: somatic complains (S3), with low *R*, social problems (S4) and total, with higher *R*. Linear regression achieves the best *R* in thought (S5, regressor lreg), internalizing (ridge), and externalizing problems (lm, with high *R*). Regression trees and ensembles provide the best prediction for anxious/depressed (S1, adaboost); attention problems (S6, pyrf); rule-breaking (S7, extraTrees); and aggressive behavior (S8, tree). Neural network is the best for withdrawn/depressed behavior (S2, pymlp).

Regarding classification into normative vs. preclinical and clinical (last three rows of Table 3), accuracy is almost perfect (97.2%) in externalizing with linear regression, lm. Except social problems (S4), the accuracy is above 70% in all the outcomes, with values close to or above 80% for anxious/depressed (S1), somatic complains (S3), attention problems (S6), rule-breaking (S7), and internalizing. Overcoming sensitivity outcomes was 80% in social (S4) and attention (S6) problems, and 100% in externalizing, being close to or above 70% in the remaining CBCL scales except anxious/depressed (S1), somatic complains (S3), rule-breaking (S7), and aggressive behavior (S8). Specificity values were 80% in anxious/depressed (S1), thought (S5), and attention (S6) problems, and 92.3% in externalizing, being close to or above 70% for withdrawn/depressed (S2), social problems (S4), and internalizing and total problems. The lowest specificity values are achieved in somatic complaints (S3), rule-breaking (S7), and aggressive behavior (S8), which are globally the worst-performing CBCL outcomes.

**Figure 1:**
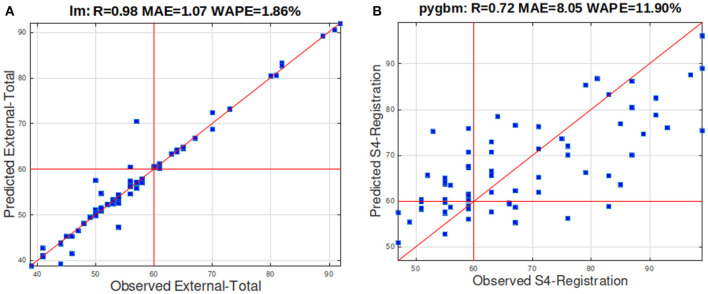
**(A)** Scatterplot of the true values and values predicted by lm (linear regression) for the externalization domain. **(B)** Scatterplot of the true values and values predicted by pygbm for social problems (S4). The *R*, MAE, and WAPE values are reported in the title.

Figure 1 plots the observed (horizontal axis) and predicted (vertical) values for externalizing (Figure 1A) and social problems (S4, Figure 1B) for all participants with the best regressor (lm and pygbm, respectively), and the best SP2 input set (total and registration, respectively). Figure 1A depicts the high coincidence between both values, with WAPE below 2%, while Figure 1B shows lower coincidence values, with WAPE around 12%, although the predicted values somehow raise with the true values, providing *R* = 0.72 (qualified as “moderate to good”). The CBCL values (blue squares) depicted on the right (left) of the red vertical line at 60 are participants who scored in the preclinical and clinical (in the normative) range. Likewise, the squares above (below) the horizontal red line at 60 are participants who are the best regressors predicted in the preclinical and clinical (in the normative) range. All the squares on the upper right and lower left areas defined by the horizontal and vertical red lines at 60 are predicted as the right class label, and only values on the upper-left and lower-right quadrants are predicted as the wrong class label. Thus, the number of classification errors is very low in panel A (externalization domain), with no false negatives (dots in the lower right quadrant, 100% sensitivity) and just two false positives (upper left quadrant). In panel B, the number of dots in the upper left and lower right quadrants is higher, but much lower than in lower left and upper right quadrants (patients well-classified), with an accuracy of 66.7%, so the impact of unreliability in prediction (*R* = 0.72) over classification is low for this outcome.

## Discussion

This study investigated how sensory processing alterations predicted behavior problems in children and adolescents with ASD using ML models. This technique offers a new potential solution to classify ASD difficulties based on different dimensions, e.g., behavior, genetics, and neuroimaging data, among others (Georgescu et al., 2019). These artificial intelligence tools can be an important and useful approach to further explain how sensory processing abnormalities (one of the earliest clinical alterations in ASD) are predictive of social, behavior, and emotional problems in this population.

Overall, results revealed high correlation between some true behavioral (CBCL) scales and the values predicted by the regressors using some SP2 processing quadrants. The highest correlation was observed for total problems, anxiety/depression, social problems, and externalizing scales. The prediction was more reliable using certain SP2 scales, revealing strong relations with the predicted CBCL scales: CBCL externalizing problems and SP2 total scale score; CBCL social problems and SP2 registration quadrant; CBCL anxiety/depressed and SP2 avoiding quadrant; and CBCL total problems scale with SP2 touch scale. With this prediction, early indicative signs of the overall behavioral difficulties of children with an ASD diagnosis can be obtained from their response to sensory stimuli. Thus, these results can be extremely useful for clinical interventions. Considering the predicted information, early signs of altered sensory functioning would allow for incorporating personalized and early sensory-based care therapies for children with an ASD diagnosis, hence minimizing the affected areas of behavior, as much as possible.

Results obtained by the ML methods are consistent with other findings, suggesting an association between sensory processing and behavior problems in children and adolescents with ASD (Iarocci and McDonald, 2006). Patterns of sensory processing alterations, including touch processing, may imply difficulties in response to environmental cues and missing opportunities to learn from them, which is the foundation for the development of more complex processes, such as emotional regulation, and social interactions (Kojovic et al., 2019). These difficulties have cascade effects in daily life activities in ASD, manifested through a range of behavior problems such as hyperactivity, impulsivity, stereotyped and repetitive behaviors, as well as emotional and social distress (Thye et al., 2018). It may contribute to the emergence of anxious and depressive states, as dealing with sensory stimulation may be demanding for children and adolescents with ASD (Fernández-Prieto et al., 2021).

The results contribute to the existing evidence highlighting the detrimental impact of altered sensory processing on behavioral outcomes in children and adolescents with ASD (Foss-Feig et al., 2012; Uljarević et al., 2016; Miguel et al., 2017). These difficulties interfere with their adaptive functioning (McLean et al., 2014) and should be considered in interventional approaches with this population. Early intervention programs focused on addressing sensory difficulties may help prevent behavioral problems, alleviate behavioral difficulties, and, consequently, improve adaptive functioning of children and adolescents to environmental situations.

This work offers multiple strengths, of which how ML methods contribute to clarify the association between sensory processing impairments and behavioral difficulties in children and adolescents with ASD is highlighted, as these results can provide important clues for sensory-related intervention approaches within this population. However, this study has some limitations. First is the reduced number of participants. Future studies should consider a larger sample to confirm the results. In addition, it would be important to replicate this investigation in ASD adult population to verify if similar outcomes are present. Also, it would be important that future studies address severity levels of ASD presentations, considering the high inter-individual variability observed in this population. Another limitation of this work refers to its cross-sectional design that does not allow for determining causal associations between the variables. Thus, future studies should consider assessing sensory processing and behavioral outcomes in a longitudinal manner to clarify the causal relationship between these dimensions. Also, future investigations should integrate brain imaging techniques to elucidate about the areas of the brain that pertain to sensory processing and/or behavior that are affected, which would strengthen these findings.

## Conclusion

Autism spectrum disorders are associated with sensory processing abnormalities that often lead to behavioral alterations. The current study investigates the relations between the SP2 scales and the CBCL outcomes in order to study the association between sensory and behavior problems, using 26 machine learning regressors of different families: linear regression, kernel and support vector regression, ensembles including bagging, adaboost, gradient boosting machine and random forest, regression trees, and neural networks. Using a sample of 72 participants, the predicted outcomes are “from good to excellent” for externalization domain using all the SP2 items, and “from moderate to good” for anxious/depressed and social problems using avoiding and registration quadrants, respectively. The predictions are also “from moderate to good” for the remaining outcomes except somatic complaints and rule-breaking, where the predictions are “bad to moderate.” Considering the classification into normative vs. preclinical and clinical, the accuracy reaches 97.2 and 87.5% for externalization and anxious/depressed, respectively. However, somatic complaints and rule-breaking outcomes, with low predictive reliability, still achieve accuracies near 80%, alongside with attention problems and internalization, while the remaining outcomes achieve accuracies above or near 70%.

## Data Availability Statement

The original contributions presented in the study are included in the article/Supplementary Materials, further inquiries can be directed to the corresponding author.

## Ethics Statement

The studies involving human participants were reviewed and approved by the Research Ethics Committee of Santiago-Lugo. Written informed consent to participate in this study was provided by the participants' legal guardian/next of kin.

## Author Contributions

HA: article writing and experimental work with machine learning regressors. SC: article writing. EC: analysis of regression results. MT-F: conceptualization, methodology, investigation, and writing. AS: discussion of results in ASD context. AG-V: discussion of relations between sensory and behavior. AC: resources, writing, review, editing, and supervision. MF-D: execution of regression models. MF-P: writing of original draft and review, visualization, and project administration. All authors contributed to the article and approved the submitted version.

## Funding

This work has received financial support from the Consellería de Educación, Universidade e Formación Profesional (accreditation 2019-2022 ED431G-2019/04) and the European Regional Development Fund (ERDF), which acknowledges the CiTIUS—Centro Singular de Investigación en Tecnoloxías Intelixentes da Universidade de Santiago de Compostela as a Research Center of the Galician University System. SC acknowledges the Centro de Investigação em Psicologia para o Desenvolvimento (CIPD) [The Psychology for Positive Development Research Center] (UID/PSI/04375), Lusíada University North, Porto, supported by national funds through the Portuguese Foundation for Science and Technology, I.P., and the Portuguese Ministry of Science, Technology, and Higher Education (UID/PSI/04375/2019). AS was supported by the Psychology Research Center (PSI/01662), School of Psychology, University of Minho, through the Foundation for Science and Technology (FCT) through the Portuguese State Budget (Ref.: UIDB/PSI/01662/2020). MT-F, AC, and MF-P were funded by Instituto de Salud Carlos III (PI19/00809 to AC) and co-funded by European Union (ERDF) A way of making Europe, and Fundación María José Jove.

## Conflict of Interest

The authors declare that the research was conducted in the absence of any commercial or financial relationships that could be construed as a potential conflict of interest.

## Publisher's Note

All claims expressed in this article are solely those of the authors and do not necessarily represent those of their affiliated organizations, or those of the publisher, the editors and the reviewers. Any product that may be evaluated in this article, or claim that may be made by its manufacturer, is not guaranteed or endorsed by the publisher.

## Footnotes

^1^We used several implementations of linear regression because they achieved different results.
