# Supplementary material for: A Machine Learning Approach in Autism Spectrum Disorders: From Sensory Processing to Behavior Problems
Source: Front Mol Neurosci. 2022 May 9;15:889641. doi: 10.3389/fnmol.2022.889641 (PMC9126208; doi:10.3389/fnmol.2022.889641)
Supplement: Supplementary file 1 [file Data_Sheet_1.PDF]

Supplementary Material

1 Sensory Profile – 2

The sensory profile – 2 (Dunn, 2014) 3:0-14:11, Spanish version, measures children and adolescents' sensory processing abilities. It is a parent-report questionnaire that consists of 86 items scored on a five-point *Likert*-type scale (1 = "Almost Never", 2 = "Occasionally", 3 = "Half of the time", 4 = "Frequently", 5 = "Almost Always"). Sensory modalities include Auditory Processing, Visual Processing, Touch Processing, Movement, Body Position and Oral Processing, while Behavioral sections include Conduct, Social-emotional and Attentional. Total scores allow to characterize the sensory processing response into four different quadrants: Seeking (more engaged in sensory experiences and looks to increase sensory input), Avoiding (retreats from unfamiliar situations and/or avoids or limits sensory experiences), Sensitivity (reacts more intensely or responds to sensory stimuli that are not detected by others) and Registration (reacts later to sensory experiences or does not respond to sensory stimuli that other people usually respond to). For the purposes of this study, the four quadrants, the Touch processing sensory section score, and the Total score (the sum of all quadrants) were used.

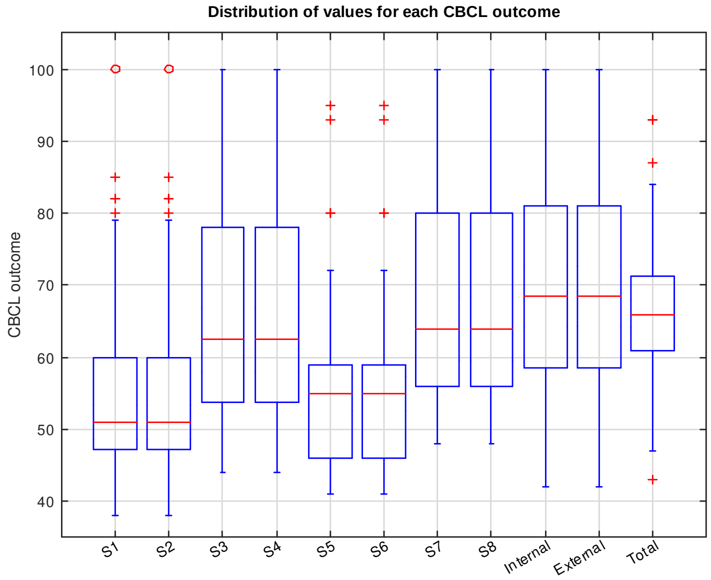

Figure 1: Box plots of the 11 CBCL outcomes to be predicted. The blue box represents the 25%-75% percentiles, the red horizontal line is the median, the blue whiskers are the data outside this interval and the red crosses and circles are outliers.

2 Child Behavior Checklist

The Child Behavior Checklist (Achenbach and Rescorla, 2001), CBCL/6-18, Spanish version, was used to assess emotional, behavioral, and/or social problems in school age children and adolescents from 6 to 18 years. This is a parent-report questionnaire that consists of 113 items rated on a three-point Likert-type scale (0 = Not True (as far as you know), 1 = Sometimes or Somewhat True, or 2 =

Very True or Often True). The following scales are obtained: anxious/depressed (S1), withdrawn/depressed (S2), somatic complaints (S3), social problems (S4), thought problems (S5), attention problems (S6), rule-breaking (S7), and aggressive behavior (S8). The sum of S1, S2, and S3 scales allows to obtain the internalizing domain; the sum of S7 and S8 scales gives the externalizing domain. The "total problems" domain is achieved by summing internalizing, externalizing and "other problems". Figure 1 plots the boxplots with the values of these 11 CBCL outcomes.

3 Autism Diagnostic Interview Revised

The Autism Diagnostic Interview Revised, ADI-R (Rutter, 2006) is a clinical interview useful for the ASD diagnosis. It is focused on the assessment of core symptoms of the disorder, including sensory processing. This parent-report questionnaire consists of 93 questions focused on three main domains related to ASD diagnosis: language/communication, reciprocal social interactions and restricted, repetitive and stereotyped behaviors and interests. Questions are focused on the clinical history of the patient and their current situation (recent months prior to the assessment).

4 Autism Diagnostic Observation Schedule – 2

The Autism Diagnostic Observation Scale - 2 ADOS-2, (Lord, 2008) is used to assess clinical traits of ASD - communication, social interaction and play or imaginative use of materials. It consists of five modules (T, 1, 2, 2, 3 and 4) that are applied according to the language skills and chronological age of the patient. Each module is composed of a set of activities and, according to patient's performance, the evaluator observes and assess the presence of social and communicative behaviors relevant to the ASD diagnosis.

5 Machine learning techniques

Machine learning is an important topic in artificial intelligence that encloses, among others, supervised methods (or models) for the automatic prediction of continuous (regression) or discrete (classification) values based on data examples. A model learns to predict the outcome value as a function of the input data in a process called "training", that uses a collection of examples, each composed by the input data and the outcome value. During training, the model changes the values of its parameters in order to predict an outcome near to the true value for the training data, i.e., to give a reliable prediction for these data. The trained model is expected to generalize its predictions with reliability to new input data not used during training. In this study, the outcomes are the scale scores of the CBCL questionnaire, that have continuous values, so their prediction is a problem of "regression" and the models used to predict them are named "regressors". Often, regressors have hyper-parameters, i.e. values that are not calculated during training but they must be set previously, that influence the reliability of the model prediction. Their values are usually set by trying several values and selecting the one with the highest reliability (see subsection "Experimental methodology") on a separate dataset, in a process named "hyper-parameter tuning". Tables 1 and 2 list the regressors used in the current study, the language and package or function used to execute each regressor, its hyper-parameters and the values used for their tuning in order to achieve a good performance for each dataset. For the regressors implemented in the R statistical computing language, the collection of hyper-parameter values used by each regressor and dataset was provided by the getmodelInfo function of the caret R package (Kuhn, 2022). Figure 2 shows the boxplots of the R values achieved by each regressor for all the 132 datasets. The maximum R value is near 1 for lm in one dataset (upper red cross), and about 0.6-0.7 for the remaining regressors (upper blue segments).

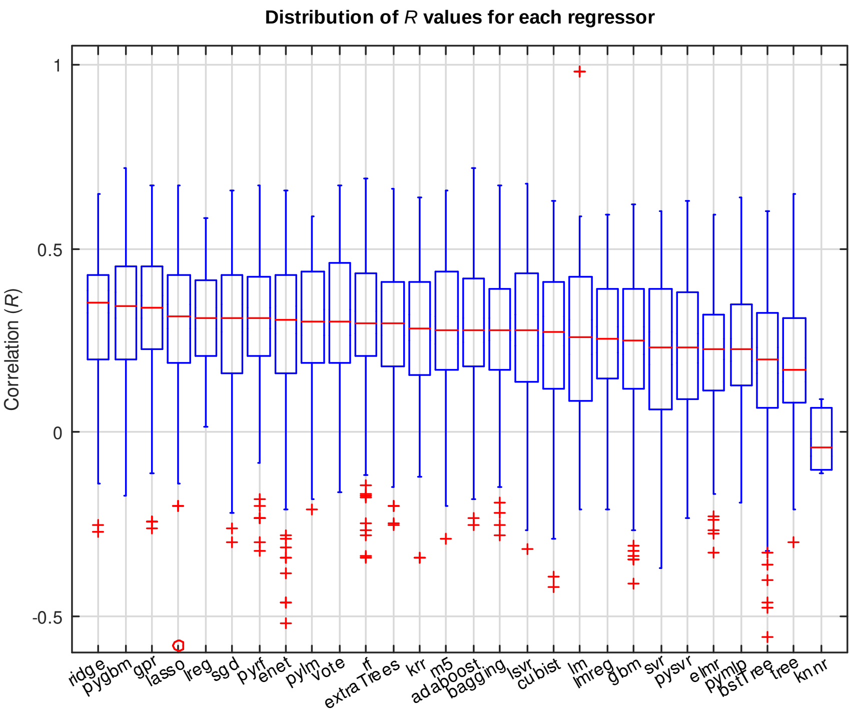

Figure 2: Box plots of the *R* values achieved by each regressor (red line is the median) over all the SP2 item sets and CBCL outcomes.

Table 3 reports the Friedman rank (Sheskin, 2006) of the regressors over the 132 datasets, sorted by decreasing performance. The rank of a regressor is its average position over the 132 datasets in the list of regressors sorted from the best to the worst, so the lower rank, the more reliable prediction. The best regressors are ridge, with rank 8.2, and gpr (8.5). Among ensembles, pygbm is the best (9.9), outperforming gbm (16.7). All the linear models (lasso, lreg, pylm, lm, enet, sgd, krr and lmreg) achieve ranks in 10-14. Other good ensembles are vote (10.7), random forests (pyrf and rf, about 11) and adaboost (13). Surprisingly, the linear kernel support vector regression (lsvr) outperforms the RBF kernel version (svr). The bad ranks of m5 (13.9) and cubist (15.5) are also surprising, given their good performance in (Fernández-Delgado et al, 2019).

6 Performance measurements

In regression problems the most popular measurements of the model performance (i.e., for the reliability of the prediction issued by the model) are the Pearson correlation coefficient (denoted as *R*), root mean squared error (RMSE), mean absolute error (MAE) and weighted absolute percentage error (WAPE), defined as:

$$R=\frac{\sum_{i=1}^{N}\left(y_i-y\right)\left(o_i-o\right)}{\sqrt{\left[\sum_{i=1}^{N}\left(y_i-y\right)^2\right]\left[\sum_{i=1}^{N}\left(o_i-o\right)^2\right]}} \qquad RMSE=\sqrt{\frac{1}{N}\sum_{i=1}^{N}\left(y_i-o_i\right)^2}$$

$$MAE = \frac{1}{N}\sum_{i=1}^{N}\left|y_i - o_i\right| \qquad WAPE(\%) = \frac{100\sum_{i=1}^{N}\left|y_i - o_i\right|}{\sum_{i=1}^{N} y_i}$$

Here, N is the number of participants, y_i and o_i are, respectively, the true outcome value and the value predicted by the regressor for the i-th participant, while y and o are the average values of y_i and o_i over the N participants, respectively. The correlation R evaluates the linear dependence between true and predicted values, scaled by their variances. The classical definition (Colton, 1974) for the significance of the correlation intervals is: $0 \leq R < 0.15$ means that true and predicted values are "not correlated at all"; $0.15 \leq R < 0.5$ means "bad to moderate correlation"; $0.5 \leq R < 0.75$ means "moderate to good correlation"; and $R \geq 0.75$ means "very good to excellent correlation". The RMSE measures the squared differences between true and predicted values, penalizing the large differences because they give larger squared differences. The MAE is the average of the absolute value of the difference between true and predicted outcomes over the N participants, so the true value is expected to be within the interval defined by the predicted value plus/minus twice the MAE. The WAPE measures the absolute difference as a percentage of the true value.

Regarding classification into normative (negative) and pre-clinical or clinical (positive) cases, the usual performance measurements are the classification accuracy (ACC), sensitivity (Se) and specificity (Sp), defined in % as:

$$ACC(\%) = \frac{100(TN + TP)}{TN + FP + FN + TP} \qquad Se(\%) = \frac{100\,TP}{FN + TP} \qquad Sp(\%) = \frac{100\,TN}{TN + FP}$$

The TP is the number of true positives, i.e., the number positive cases that the model predicts as positive; FP means false positives, the number of negative patients (normative cases) predicted as positive by the model; TN means true negatives, the number of negative patients predicted as negative by the model; and FN means false negatives, the number of positive patients predicted as negative by the model.

References

Dunn, W. (2014) Sensory profile 2 manual. San Antonio, TX: Pearson: The Psychological Corporation.

Achenbach, T.M., and Rescorla, L.A. (2001) Manual for the ASEBA school-age forms & profiles: an integrated system of multi-informant assessment. Burlington, VT: University of Vermont. Research Center for Children, Youth, & Families.

Rutter, M., Le Couteur, A., and Lord, C. (2006). ADI-R: entrevista para el diagnóstico del autismo-revisada. Madrid: TEA Editions.

Lord, C., Rutter, M., DiLavore, P.C., Risi, S., Gotham, K., Bishop, S.L., Luyster, R.J. and Guthrie, W. (2008). ADOS. Escala de observación para el diagnóstico del autismo. Madrid: TEA Editions.

Kuhn, M. (2022). Caret: classification and regression training. R package, http://topepo.github.io/caret [Accessed March 3, 2022].

Sheskin, D. (2006) Handbook of parametric and nonparametric statistical procedures. Boca Raton (FL): Chapman and Hall/CRC Press.

Fernández-Delgado. M., Sirsat, M.S., Cernadas, E., Alawadi, S., Barro, S., and Febrero-Bande, M. (2019) An extensive experimental survey of regression methods, *Neural Netw*. 111, 11-34. doi: 10.1016/j.neunet.2018.12.010

Colton, T. (1974) Statistics in medicine. Little Brown and Co.

Table 1: List of the 26 regressors executed, grouped by families, alongside with the implementation language, package or function used, hyper-parameters and values used for tuning (the notation 1:2:10 means values from 1 to 10 with step 2). All the regressors implemented in Python used the scikit-learn module.

Regressor	Language	Description	Package/function/hyper-parameter tuning
Linear and regularized linear regression			
lm	R	Linear regression	stats package
lreg	Octave	Linear regression	LinearRegression function
lmreg	Matlab	Linear regression	fitlm function
pylm	Python	Linear regression	linear_model.LinearRegression function
ridge	Python	Regularized ridge regression	linear_model.Ridge function<br>Regularization α=0:20:500
sgd	Python	Stochastic gradient descent	linear_model.SGDRegressor function<br>Regularization α=0.0001:0.5 (10 val)
lasso	Python	Regularized regression using least absolute shrinkage and selection operator (LASSO)	linear_model.Lasso function<br>Regularization α=-1:0.2:3
enet	Python	Elasticnet	linear_model.Elasticnet function<br>Regularization α=0.1:0.5:10
Kernel and support vector regression			
krr	Python	Kernel ridge regression (radial basis function kernel)	linear_model.Elasticnet function, regularization α=0.1:0.5:2, kernel spread $\sigma=2^{-10:2:10}$
svr	Octave	ε-support vector regression with radial basis function kernel,	LibSVM library: svmtrain/svmtest functions;<br>Regularization $\alpha=2^{-5:2:15}$, kernel spread $\sigma=2^{-10:2:10}$
lsvr	Octave	Linear ε-SVR, LibSVM	Regularization $\alpha=2^{-5:2:15}$
gpr	Python	Gaussian process regression	gaussian_process.GaussianProcessRegressor<br>gaussian_process.kernels.DotProduct,WhiteKernel
pysvr	Python	ε-support vector regression with radial basis function kernel	svm.SVR function. Regularization $\alpha=2^{-5:2:15}$<br>kernel spread $\sigma=2^{-10:2:10}$
Regression trees			
m5	R	M5 model tree	RWeka package
tree	Python	Regression tree	tree.DecisionTreeRegressor function

Table 2: Continuation of Table 1.

Regressor	Language	Description	Package/function/hyper-parameter tuning
Ensembles			
bagging	Python	Bagging	ensemble.BaggingRegressor function<br>No. estimators=10:5:50
adaboost	Python	Adaboost.R2	ensemble.AdaBoostRegressor function<br>No. estimators=10:10:50
gbm	R	Gradient boosting machine	gbm package, depth=1:10; ntrees=50:50:500
pygbm	Python	Gradient boosting machine	ensemble.GradientBoostingRegressor function<br>No. trees=50,100,150,200; Depth=1,3,6,9
bstTree	R	Boosting ensemble	bst package
cubist	R	Boosting of regression trees with nearest neighbor correction	Cubist package; no. neighbors=0,5,9<br>no. committees=1,10,20
rf	R	Random forest	RandomForest package<br>mtry=2,..,number of SP2 scores (10 values)
pyrf	Python	Random forest	ensemble.RandomForestRegressor function<br>max. features=2,..,number of SP2 scores (10 values)
extraTrees	R	Extremely randomized regression trees	extraTrees package
vote	Python	Voting committee of linear regression and random forest	ensemble.VotingRegressor, LinearRegression, RandomForestRegressor
Neural networks			
pymlp	Python	Multi-layer perceptron	neural_network.MLPRegressor function<br>No. hidden neurons=10:20:200

Table 3: Friedman rank (decreasing with the reliability in prediction) of the regressors over the 132 combinations of SP2 item sets and CBCL outcomes with and without gender.

Position	Regressor	Family	Rank	Position	Regressor	Family	Rank
1	ridge	Linear	8.2	14	krr	Linear	13.4
2	gpr	Kernel	8.5	15	lmreg	Linear	13.5
3	pygbm	Ensemble	9.9	16	lsvr	Support	13.6
4	lasso	Linear	10.4	17	extraTrees	Ensemble	13.7
5	lreg	Linear	10.6	18	m5	Tree	13.9
6	vote	Ensemble	10.7	19	bagging	Ensemble	14.3
7	pyrf	Ensemble	11.6	20	cubist	Ensemble	15.5
8	rf	Ensemble	11.8	21	pymlp	Network	16.3
9	pylm	Linear	12.8	22	gbm	Ensemble	16.7
10	lm	Linear	12.9	23	pysvr	Kernel	17.4
11	adaboost	Ensemble	13.0	24	svr	Kernel	17.5
12	enet	Linear	13.1	25	bstTree	Ensemble	18.7
13	sgd	Linear	13.2	26	tree	Tree	19.0
